# Progress and implementation of team-based care in the United States

**DOI:** 10.1186/1472-6963-14-S2-P46

**Published:** 2014-07-07

**Authors:** Debora Goldberg, Anton Kuzel

**Affiliations:** 1 Department of Health Policy, George Washington University, Washington DC, USA; 2 Department of Family Medicine and Population Health, Virginia Commonwealth University, Richmond VA, USA

## Background

There are currently many changes taking place in the health care system in the United States. Major transformations occurring in primary care settings strive to improve efficiency and effectiveness of health promotion and prevention activities, coordination of patient care, and management of chronic diseases. Team-based care is a major component of primary care redesign.

The purpose of this study was to gain an in-depth understanding of how primary care practices in the United States are implementing team-based care. The study focused on understanding team-based care approaches, challenges to implementation, and successful strategies.

## Materials and methods

A multidisciplinary research team used qualitative research methods to conduct in-depth case studies of eight primary care practices in various stages of practice transformation. The research team collected data from practices over a sixteen-month period using on-site interviews and structured telephone questionnaires (N=51), observation, and document review. In addition, key informant interviews were conducted with sixteen (N=16) leaders of primary care delivery organizations across the United States to obtain detailed information on team-based care models, such as team composition, roles and responsibilities of team members, and how teams function to meet the needs of their patients.

## Results

The composition of teams and team member functions vary greatly across health care organizations. Team members typically include one or more providers (physician, physician assistant or nurse practitioner), registered nurses (RN) or licensed practical nurses (LPN), medical assistants (MA), and administrative staff. In some settings teams include pharmacists, behavioral health specialists, social workers, community health workers, patient navigators and/ or care coordinators. Many organizations recognize the patient as an important member of the team.

Organizational culture, values set by practice leaders, and other factors influence implementation of team-based care. Successful strategies for implementation include: leadership support and commitment, a multidisciplinary design and implementation team, careful review and redesign of processes, communication through formal policies and procedures and regular team meetings, and collection and review of performance data. Culture for team-based care models center on shared values and respect for individual team members’ skills and contribution.

## Conclusion

The future of primary care in the United States will be characterized by many models of care that incorporate different types of health care professionals. Team-based care is a promising method for improving care coordination, medication management, patient education and self-management, and increasing the delivery of preventive services. Team-based care models may also improve productivity and boost employee morale.

